# Comparison of mortality and survival without major morbidities of very preterm infants with very low birth weight from Japan and Brazil

**DOI:** 10.1590/1984-0462/2023/41/2021389

**Published:** 2022-09-09

**Authors:** Caroline Kaori Tomo, Olukunmi Omobolanle Balogun, Josy Davidson, Ruth Guinsburg, Maria Fernanda Branco de Almeida, José Maria de Andrade Lopes, Marina Carvalho de Moraes Barros, Kenji Takehara, Masashi Mikami, Tetsuya Isayama, Ai Hoshino, Rintaro Mori, Masashi Mizuguchi

**Affiliations:** a University of Tokyo, Tokyo, Japan.; b National Center for Child Health and Development, Tokyo, Japan.; c Universidade Federal de São Paulo, São Paulo, SP, Brazil.; d Kyoto University, Kyoto, Japan.

**Keywords:** Neonatal sepsis, Mortality, Morbidity, Infant, Very low birth weight, Infant, extremely premature, Sepse neonatal, Mortalidade, Morbidade, Recém-nascido de muito baixo peso, Lactente extremamente prematuro

## Abstract

**Objective:**

This study was carried out to understand the disparities in mortality and survival without major morbidities among very premature and very low birth weight infants between participating Neonatal Intensive Care Units (NICUs) from the Brazilian Network on Neonatal Research (RBPN) and the Neonatal Research Network of Japan (NRNJ).

**Methods:**

Secondary data analysis of surveys by the RBPN and NRNJ was performed. The surveys were conducted in 2014 and 2015 and included 187 NICUs. Primary outcome was mortality or survival without any major morbidity. Logistic regression analysis adjustment for confounding factors was used.

**Results:**

The study population consisted of 6,406 infants from the NRNJ and 2,319 from the RBPN. Controlling for various confounders, infants from RBPN had 9.06 times higher adjusted odds of mortality (95%CI 7.30–11.29), and lower odds of survival without major morbidities (AOR 0.36; 95%CI 0.32–0.41) compared with those from the NRNJ. Factors associated with higher odds of mortality among Brazilian NICUs included: Air Leak Syndrome (AOR 4.73; 95%CI 1.26–15.27), Necrotizing Enterocolitis (AOR 3.25; 95%CI 1.38–7.26), and Late Onset Sepsis (LOS) (AOR 4.86; 95%CI 2.25–10.97).

**Conclusions:**

Very premature and very low birth weight infants from Brazil had significantly higher odds for mortality and lower odds for survival without major morbidities in comparison to those from Japan. Additionally, we identified the factors that increased the odds of in-hospital neonatal death in Brazil, most of which was related to LOS.

## RESUMO

**Objetivo:**

Este estudo foi realizado para compreender as disparidades na mortalidade e sobrevivência sem as principais morbidades entre recém-nascidos muito prematuros e de muito baixo peso entre Unidades de Terapia Intensiva Neonatal (UTINs) participantes da Rede Brasileira de Pesquisas Neonatais (RBPN) e Rede de Pesquisa Neonatal do Japão (NRNJ).

**Métodos:**

Foi realizada uma análise dos dados secundários dos bancos de dados da RBPN e da NRNJ. As pesquisas foram realizadas em 2014 e 2015 e incluíram 187 UTINs. O desfecho primário foi mortalidade ou sobrevida sem qualquer morbidade importante. Utilizou-se a análise de regressão logística com ajuste para os fatores de confusão.

**Resultados:**

A população do estudo foi composta por 6.406 recém-nascidos do NRNJ e 2.319 do RBPN. Ajustando para diversos fatores de confusão, os prematuros da RBPN tiveram 9,06 vezes maiores chances de mortalidade (IC95% 7,30–11,29) e menores chances de sobrevivência sem morbidades importantes (AOR 0,36; IC95% 0,32–0,41) em comparação com os da NRNJ. Fatores associados a maiores chances de mortalidade entre as UTINs brasileiras incluíram: síndrome de escape de ar (AOR 4,73; IC95% 1,26–15,27), enterocolite necrosante (AOR 3,25; IC95% 1,38–7,26) e sepse de início tardio (AOR 4,86; IC95% 2,25–10,97).

**Conclusões:**

Os recém-nascidos muito prematuros e de muito baixo peso do Brasil apresentaram chances significativamente maiores de mortalidade e menores chances de sobrevivência sem as principais morbidades em comparação aos do Japão. Além disso, identificamos os fatores que aumentam as chances da morte neonatal no Brasil, sendo a maioria relacionada à sepse tardia.

## INTRODUCTION

There have been many technological and therapeutic advances in perinatal care over the past several decades globally. Regardless, there is still significant room for improvement in the healthcare management for very premature (VPT) and very low birth weight (VLBW) infants, especially with regard to preventing and reducing neonatal morbidity and mortality rates.^1^

Despite the decline rate in neonatal mortality globally, marked disparities exist across regions and between countries.^2^ Brazil, the largest country in South America, is an upper middle-income country, characterized by vast economic and social inequalities. It is among the top 10 countries with the highest number of preterm births,^3^ and monitoring infant mortality is a priority for the country. Neonatal mortality has been the main component of child mortality since the 1990s and remained at high levels with a rate of 8 deaths per 1,000 live births in 2018.^4,5^ Most neonatal deaths occur during the early neonatal period and are associated with prematurity, congenital anomalies, intrapartum asphyxia, perinatal infections, and maternal factors. A considerable proportion of these deaths are preventable by proper action of health services.^6^ A child born in Brazil is more likely to die in the first month of life compared to one born in a high-income country such as Japan.^5^ Japan is recognized for having a remarkably low mortality rate, especially in neonates born at 25 weeks of gestational age (GA),^7^ and is rated as the country with one of the lowest neonatal mortality rate in the world.^5^

Mortality or survival without major morbidities are indicators for assessing the quality of perinatal care within a country, and to improve the quality of perinatal care, it is important for health outcomes of newborns to improve.

This study was designed to highlight important differences between Brazil and Japan in terms of neonatal health outcomes and to identify best practices and interventions that have been effective in decreasing neonatal mortality. Here, we compared rates of mortality and survival without major morbidities in VPT and VLBW infants between participating neonatal intensive care units (NICUs) from the Brazilian Network on Neonatal Research (*Rede Brasileira de Pesquisas Neonatais* — RBPN) and the Neonatal Research Network of Japan (NRNJ). So far, no comparison between Japan and Brazil had been made. This study will highlight significant disparities between both countries with regards to these outcomes, and provide an understanding into which factors are related to the differences observed. Understanding the reasons behind the disparities between the outcomes for VPT and VLBW infants in Brazil compared to Japan, a reference country in neonatal health management, is a unique opportunity to share experiences on policies and best practices that have been effective in decreasing neonatal mortality.

## METHOD

Brazilian Network on Neonatal Research: The Brazilian Network on Neonatal Research was established in 1999. The network includes 20 university hospitals located in 15 municipalities across seven Brazilian states that provide healthcare services to public patients from the Brazilian Unified Health System.^8^ Brazil operates a mixed health system, composed of two sectors: (1) a large public sector that offers universal health coverage — under which antenatal, intrapartum, postnatal, and neonatal care services are provided free of charge to all citizens enrolled in the national health scheme and; (2) a growing private sector, that includes a supplementary health care system which, according to data from the Ministry of Health, covers up to 30% of all Brazilian citizens.^9^

Neonatal Research Network of Japan: The Neonatal Research Network of Japan was established in 2003 with a grant from the Ministry of Health, Labor, and Welfare in Japan, and consists of records on VLBW infants (birth weight less than or equal to 1500g) who were born in or transferred to the participating hospitals within 28 days of birth.^10^ This multicenter registry is maintained by the NRNJ as a Non-Profit Organization. Japan's health system is characterized by the universal health insurance scheme, and participants are free to choose healthcare facilities from which they receive care.

This was a retrospective cohort study that included all infants born at 26 through 32 weeks GA and with birth weight <1500g from January 2014 to December 2015 who were admitted to NICUs in the NRNJ (167 NICUs) or RBPN (20 NICUs). Gestational and neonatal data were analyzed from delivery until death or discharge from the NICU. Infants with congenital anomalies or those who were moribund on admission (where a decision was made at birth not to provide resuscitative care) were excluded from the study.

Maternal and infant data in both networks were collected using data forms based on the international neonatal database of VLBW infants from the Vermont Oxford Network.^11^ Using this standardized form allowed us to obtain many variables in common between the two networks. The variables used to describe maternal and infant characteristics included: gender, birth weight, GA, Apgar score,^12^ type of delivery (cesarean or vaginal delivery), mother's age, complications during pregnancy (gestational diabetes mellitus, hypertension during pregnancy, clinical chorioamnionitis), multiple births, and antenatal steroid use.

Gestational age was compiled following the method of Naegele^13^ and Ballard^14^ in weeks and was determined by the date of the last menstrual period, followed by the first-trimester ultrasound and, if both were unavailable, by physical examination of the newborn. Antenatal steroid use was defined as administration of at least one dose of corticosteroid to the mother at any time before delivery to accelerate fetal lung maturity.

The main outcomes in this study were mortality before the last discharge from NICU and survival without any of the four major morbidities: bonchopulmonary dysplasia (BPD), severe neurological injuries (SNI; intraventricular hemorrhage grades III or IV or periventricular leukomalacia), and necrotizing enterocolitis (NEC). These morbidities were chosen because they have been reported to be related to long-term physical and neurodevelopmental disabilities with higher chances of mortality.^15–19^

We have also made a consensual definition of all other outcome variables from both networks due to differences in the definitions used by each network defined in a consultative process based on available data and expert neonatologists’ opinions. The definition of Mortality, SNI and NEC was maintained since both neonatal networks have the same definition. Moreover, RBPN considers BPD as the requirement for inspired oxygen at a fraction above 0.21 at a corrected GA of 36 weeks; and NRNJ acknowledges BPD as oxygen use at 36 weeks corrected GA with oxygen use on 28^th^ day after birth. The consensus was that the definition of BPD would be oxygen use at 36 weeks corrected GA with fraction above 0.21.

A comparison between maternal and infant characteristics from the two networks was carried out using descriptive statistics. Mean and standard deviation were used for continuous variables and frequency and percentage for categorical variables. The number of infants deaths, survival without major morbidities, infants with BPD, SNI, NEC, sepsis, patent ductus arteriosus (PDA), and air leak syndrome was compared using the chi-square test. It was considered significant if p values were p<0.001.

Multiple logistic regression analysis was done to analyze the differences in mortality (before discharge) and survival without major morbidities between the two networks. The model was adjusted for all variables related to conditions before birth. The adjustment variables in the model included maternal age (categorized as <20, 20–34, and <35 years of age), gestational diabetes mellitus (GDM), maternal hypertension, chorioamnionitis, prenatal steroid, cesarean delivery, gender, GA, Apgar score at 1 minute and multiple births. Infant birth weight correlated strongly with GA and was therefore not included in the model.

To further identify the risk factors of neonatal death in Brazil, a multiple logistic regression adjusted for all the variables in the RBPN database was performed. Among the factors with collinearity, the variables with greater clinical relevance were kept. As the variables of Apgar ≤3 at 5 minutes, use of oxygen, use of invasive mechanical ventilation, incidence of infection and early onset sepsis were highly skewed, these factors were excluded from our model. Further, because BPD was defined as oxygen use at 36 weeks of corrected GA, and the analysis did not asses the risk of mortality limiting to after 36 weeks postmenstrual age, BPD was also excluded from our model. The model was adjusted for race, maternal age, mother's educational level, prenatal care assessment, maternal hypertension, gestational diabetes mellitus, chorioamnionitis, antenatal steroid use, peripartum haemorrhage, multiple gestation, caesarean delivery, GA in weeks, gender of the baby, Apgar score ≤3 at 1 minute, resuscitation, Respiratory Distress Syndrome (RDS), air leak syndrome, persistent pulmonary hypertension, pulmonary haemorrhage, surfactant use, SNI, surgical PDA, NEC, and late onset sepsis (LOS). Results of the logistic regression analyses are presented as adjusted odds ratio (AOR) with corresponding 95% confidence intervals (95%CIs). A *Receiver Operating Characteristics Curve* with its associated area under the curve (AUC) were generated to evaluate the accuracy of the final generalized regression model. Furthermore, a Kaplan-Meier survival curve was plotted in order to analyze the incidence of mortality between groups. All statistical analyses were done using R Studio statistical software (version 1.1.447).

Ethical approval for this study was obtained from the ethical committees of the NRNJ, RBPN, and University of Tokyo. Data collection by the RBPN was approved by the appropriate quality and data control committee at each institute in Brazil. The data collected by the NRNJ and the use of the studies were approved by the internal review board of the Tokyo Women's Medical University, Japan, and consent given by the respective parents or guardians. Also, the study was previously approved with CEP number 2.452.055

## RESULTS

The number of VLBW infants born alive in participating NICUs of NRNJ and RBPN between January 2014 and December 2015 was 9,254 and 2,940, respectively. After excluding infants born before 26 weeks or after 32 weeks GA (n=2,821, from NRNJ and n=617 from RBPN), infants with moribund status at birth (n=5 in NRNJ, and n=4 in RBPN), and infants in the Japanese dataset that had no information about the reason for discharge of the newborns (n=22), the final study population consisted of 8,725 infants, including 6,406 from NRNJ and 2,319 from RBPN.

A comparison of maternal and infant characteristics for participants from both networks was done and presented in Table 1. Maternal characteristics varied between the two countries, with significant differences observed in maternal age, antenatal steroid use, incidence of GDM and hypertension during pregnancy, and cesarean section (p<0.001). For infant characteristics, the mean GA at birth and birth weight were significantly higher in Japan than in Brazil (p<0.001).

**Table 1:** Comparison of the infant and maternal characteristics between Neonatal Research Network of Japan and Brazilian Network on Neonatal Research.

		NRNJ (n=6,406)	RBPN (n=2,319)	p-value
Maternal age, mean (SD)		32.3 (5.5)	27.3 (7.1)	<0.001
	<20 [n(%)]	92 (1.5)	362 (15.6)	
	20–34 [n (%)]	3,778 (61.0)	1,562 (67.4)	
	≥35 [n(%)]	2,324 (37.5)	394 (17.0)	
Antenatal steroid use [n(%)]		3,706 (61.6)	1,843 (79.5)	<0.001
Cesarean section [n(%)]		5,045 (82.1)	1,646 (71.0)	<0.001
Diabetes during gestation [n(%)]		285 (4.8)	164 (7.1)	<0.001
Hypertension [n(%)]		1,372 (23.0)	994 (43.1)	<0.001
Chorioamnionitis [n(%)]		799 (12.5)	249 (10.7)	<0.01
Multiple births [n(%)]		1,405 (21.9)	521 (22.5)	0.61
Infant characteristics		NRNJ (n=6,406)	RBPN (n= 2,319)	p-value
Male gender [n(%)]		3,303 (51.6)	1,144 (49.2)	0.07
Birth weight (g), mean (SD)		1,097.9 (260.0)	1,066.4 (254.7)	<0.001
	<500g	106 (1.8)	18 (0.8)	
	500–749g	502 (8.5)	274 (11.8)	
	750–999g	1,522 (25.7)	621 (26.7)	
	1000–1249g	1,782 (30.1)	739 (31.9)	
	1250–1500g	2,014 (34.0)	667 (28.8)	
Gestational age (wk), mean (SD)		29.0 (1.8)	28.7 (1.8)	<0.001
	26–27	1,567 (24.5)	677 (29.2)	
	28–29	2,003 (31.3)	779 (33.6)	
	30–32	2,836 (44.3)	863 (37.2)	
Apgar score <3 at 1 min [n(%)]		1,421 (22.8)	518 (22.5)	0.76
Apgar score <3 at 5 min [n(%)]		268 (4.3)	79 (3.4)	0.07

NRNJ: Neonatal Research Network of Japan; RBPN: Brazilian Network on Neonatal Research; SD: standard deviation; wk: weeks.

Table 2 shows the difference in mortality and incidence of major morbidities in all newborns and in newborns stratified by GA across both networks. Infants in the RBPN had a significantly higher mortality rate compared to those in the NRNJ (20 vs. 3.1%; p<0.001). The difference in mortality and major morbidities was inversely proportional to GA, whereby the lower the GA, the greater the difference between mortality and morbidity rates between both networks.

**Table 2:** Comparison of outcomes between Neonatal Research Network of Japan and Brazilian Network on Neonatal Research.

	Network	All VLBW	p-value	26~27 wk	p-value	28~29 wk	p-value	30~32 wk	p-value
No. of infants	NRNJ	6,406		1,567		2,003		2,836	
	RBPN	2,319		677		779		863	
Mortality	NRNJ	3.1 (198/6,406)	<0.001	5.4 (85/1,567)	<0.001	2.7 (55/2,003)	<0.001	2.0 (58/2,836)	<0.001
	RBPN	20.0 (464/2,319)		38.1 (258/678)		18.4 (143/779)		7.3 (63/863)	
Survival without major complications	NRNJ	67.8 (3,978/5,869)	<0.001	42.5 (618/1,455)	<0.001	66.3 (1,225/1,849)	<0.001	83.2 (2,135/2,565)	<0.001
	RBPN	45.7 (1,060/2,317)		23.3 (158/678)		44.2 (344/779)		64.8 (558/861)	
SNI	NRNJ	4.9 (290/5,945)	<0.001	7.5 (110/1,471)	<0.001	5.2 (97/1,863)	<0.001	3.2 (83/2,611)	<0.001
	RBPN	11.8 (271/2,290)		14.8 (97/657)		10.6 (82/772)		10.7 (92/861)	
BPD	NRNJ	19.2 (1,232/6,406)	0.34	37.6 (590/1,568)	0.58	19.4 (388/2,003)	0.47	9.0 (254/2,836)	<0.05
	RBPN	18.2 (347/1,905)		36.1 (158/438)		20.8 (136/655)		6.5 (53/812)	
NEC	NRNJ	1.1 (64/5,925)	<0.001	2.1 (31/1,465)	<0.001	1.3 (24/1,854)	<0.001	0.3 (9/2,607)	<0.001
	RBPN	7.2 (166/2,293)		9.7 (64/659)		8.8 (68/774)		4.0 (34/860)	
Sepsis	NRNJ	5.3 (313/5,940)	<0.001	10.6 (156/1,468)	<0.001	5.5 (102/1,863)	<0.001	2.1 (55/2,610)	<0.001
	RBPN	25.0 (573/2,293)		34.4 (227/659)		25.8 (200/774)		17.0 (146/860)	
PDA ligation	NRNJ	3.8 (224/5,923)	<0.01	9.2 (134/1,455)	<0.001	3.8 (71/1,858)	0.19	0.7 (19/2,610)	0.56
	RBPN	2.3 (53/2,292)		4.3 (28/658)		2.7 (21/774)		0.5 (4/860)	
Air Leak Syndrome	NRNJ	2.2 (133/5,957)	<0.001	3.6 (53/1,475)	<0.001	2.2 (41/1,862)	0.16	1.5 (39/2,620)	0.77
	RBPN	3.8 (88/2,293)		7.9 (52/659)		3.2 (25/774)		1.3 (860/11)	

The numbers indicate the percentage of incidence of infants in each network. VLBW: Very Low Birth Weight; wk, weeks; NRNJ: Neonatal Research Network of Japan; RBPN: Brazilian Network on Neonatal Research; SNI: Severe Neurologic Injury; BPD: Bronchopulmonary Dysplasia; NEC: Necrotizing Enterocolitis; PDA: Patent Ductus Arteriosus.

Rate of survival before discharge without major morbidities was 67.8% for Japanese babies compared to 45.7% among Brazilian ones. Infants in the RBPN had greater rates of SNI (11.8 vs. 4.9%; p<0.001), NEC (7.2 vs. 1.1%; p<0.001), sepsis (25 vs. 5.3%; p<0.001), and air leak syndrome (3.8 vs. 2.2%; p<0.001), but lower rates of BPD (18.2 vs. 19.2%; p=0.34) and PDA ligation (2.3 vs. 3.8%; p<0.01) compared to infants in the NRNJ.

Results of the multiple logistic regression analyses showed a significantly better performance among infants from the NRNJ compared to those from the RBPN. Infants from the RBPN had higher odds of mortality (AOR 9.06; 95%CI 7.30–11.29), and lower odds of survival without major morbidities (AOR 0.36; 95%CI 0.32–0.41) compared with their couterparts from the NRNJ.

**Figure 1:**
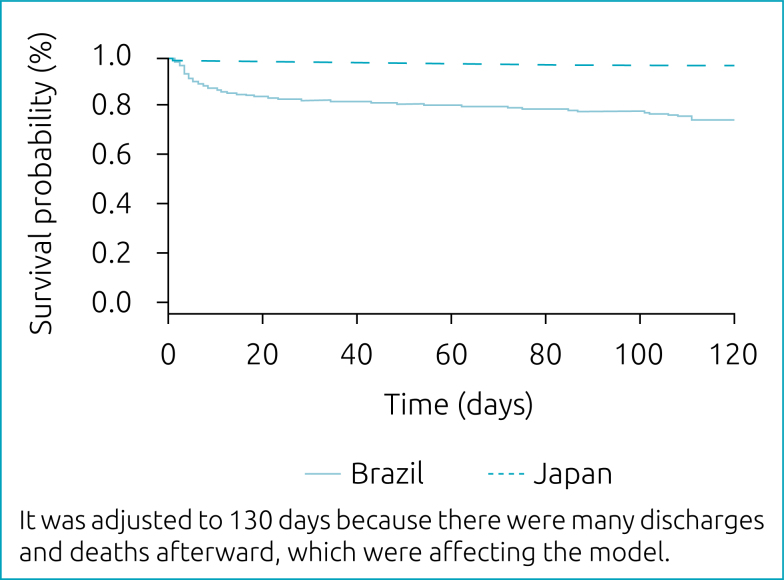
Kaplan-Meier survival curve.

The Kaplan-Meier survival curve showed significant disparity in the neonatal mortality rate between both countries (Figure 1). There was a rapid drop in survival among infants in the RBPN during the first 20 days of hospitalization. In comparison, survival rate was constant throughout the hospitalization period among infants in the NRNJ.

Factors associated with increased odds for in-hospital neonatal death among VPT and VLBW newborns hospitalized in Brazillian NICUs are shown in Table 3. Results from multiple logistic regression analysis done using data from 2,319 infants within the RBPN showed that the odds for in-hospital neonatal death increased significantly among newborns with air leak syndrome (AOR 4.73; 95%CI 1.26–15.27), NEC (AOR 3.25; 95%CI 1.38–7.26), and LOS (AOR 4.86; 95%CI 2.25–10.97) with an AUC of 0.89 (95%CI 0.85–0.93).

**Table 3:** Multiple Logistic Regression with the RBPN dataset.

Outcome: MORTALITY	
	AOR [95%CI]
Air Leak Syndrome	4.73 [1.26–15.27]
NEC	3.25 [1.38–7.26]
LOS	4.86 [2.25–10.97]

AOR: adjusted *Odds Ratio*; 95%CI: 95% confidence interval; NEC: Necrotizing Enterocolitis; LOS: Late Onset Sepsis.

Obs1: The variable birthweight was correlated with gestational age and excluded from the model. The variables 5th minute Apgar score <4, use of oxygen, use of invasive mechanical ventilation, incidence of early-onset sepsis were also excluded as they were highly skewed.

Obs 2: Maternal age categorized (reference age >20 to <35); gestational age categorized (reference 30–32 weeks of gestational age).

Results from the chi-square test between the factors found on the multiple logistic regression that are influencing neonatal mortality among the Brazilian NICUs from the RBPN showed that NEC were significantly correlated with LOS (p<0.001).

## DISCUSSION

In this retrospective cohort study, where we compared the rates of mortality and survival without major morbidities in VPT and VLBW infants from Japan and Brazil, we found that babies born in Brazil had 9.1 times higher odds of neonatal death compared to those born in Japan; while odds of survival and discharge from the NICU without significant complications (SNI, BPD, and NEC) was 2.7 times higher among VPT and VLBW infants born in Japan compared to those born in Brazil. Notably, there was a higher rate of most of the major morbidities considered in this study among infants admitted to NICUs in Brazil in comparison to Japan — SNI (Brazil: 11.8 vs. Japan: 4.9%), NEC (Brazil: 7.2 vs. Japan: 1.1%), sepsis (Brazil: 25 vs. Japan: 5.3%), and air leak syndrome (Brazil: 3.8 vs. Japan: 2.2%). The high incidence of these morbidities among Brazilian neonates may be responsible for the higher mortality rate and lower survival without major morbidities identified in Brazil in comparison to Japan, given that these morbidities are reported to be associated with higher odds of mortality among VPT and VLBW infants.^15–19^ In addition, the prevalence of pregnancy-induced hypertension was twice as high among Brazilian mothers in comparison to Japanese ones. Other studies have shown association between pregnancy-induced hypertension and adverse birth outcomes. A large-scale population-based study involving 57 million singleton live births and stillbirths (24–46 weeks) conducted in the United States found an association between pregnancy-induced hypertension and higher risks of stillbirth and neonatal mortality, especially in the latter, and higher order births.^20^

The variation observed in mortality and morbidity among VLBW neonates between networks has been reported in several studies.^7,21,22^ In contrast to a similar comparative study between two developed countries — Canada and Japan^10^ — the current study between Japan (developed country) and Brazil (developing country) showed a much higher and significant difference in mortality and survival without the main morbidities among newborns. Regardless of the overall better survival and health outcomes in the NRNJ, newborns in NICUs in Japan had higher odds for BPD. The higher odds for BPD in Japan may be due to the characteristics of the population in each unit and their inherent risks, and, more importantly, by the set of diagnostic and therapeutic interventions performed in each center. For instance, the policy for treatment of extremely premature infants with borderline viability seemed to be more aggressive in Japan than in other countries. This could result in longer ventilation time for the newborns, resulting in higher rates of BPD.^22–25^ When one compares a network of public university hospitals NICUs in a middle-income country like Brazil, with a multicenter registry network in a highly developed country like Japan, it is important to note that Brazilian neonatal network units deal daily with problems related to availability of adequate and modern equipment to support the lives of these very fragile, extremely premature infants, as well as critical staffing problems regarding ratio of patients per nurses and level of professional training of the nurse technicians responsible for these cares. It is known that both issues, especially the quality of nursing care for critically ill preterm neonates, have a profound impact on mortality.^26^

Since we were unable to obtain and compare information about care management between the two countries, we analyzed disparities in neonatal mortality rate to determine the risk factors of neonatal mortality within NICUs in the RBPN dataset. Our findings showed that neonatal mortality in Brazilian NICUs was associated with NEC, LOS, and air leak syndrome.

In the current study, within the RBPN dataset, NEC was found to be strongly correlated with LOS. NEC is the most common, life-threatening gastrointestinal emergency of preterm babies.^27^ The statistically significant correlation found between NEC and LOS is unsurprising, given that NEC is precipitated by an inflammatory cascade triggered by an inciting event or chain of events, such as utero hypoxia and sepsis.^27^ LOS was another important predictor of neonatal death in our study among Brazilian VPT and VLBW infants. A previous study in Brazil also showed that LOS was common among Brazilian VLBW infants and significantly associated with mortality. Many of the risk factors for LOS were related to clinical practices, such as nutritional practices, administration of antibiotics through invasive devices, and management of mechanical ventilation.^28^

Our study showed air leak syndrome as another important factor contributing to neonatal mortality in Brazil. Air leak syndrome is usually related to improper mechanical ventilation on premature infants’ immature and fragile lungs. According to a guideline for health care professionals from the Brazilian Ministry of Health,^29^ air leak syndrome worsens the prognosis of newborns, increasing their risk for chronic lung disease, central nervous system lesions, and mortality, especially in premature newborns. Therefore, early detection and treatment of these complications are essential, especially for neonates undergoing some ventilatory support.

Findings from the survival analysis in the current study showed a significant disparity in the neonatal mortality rate between both countries within the first 20 days of hospitalization of the newborns. Such difference could be explained by a study of Brazilian preterm infants, wherein Guinsburg et al.^9^ suggested that the outcomes of the VLBW in Brazil could be improved with better assistance in the delivery room, adequate resuscitation, minimization of hypothermia, effective ventilatory assistances, and prevention of hospital-acquired infections.

At the time of our analysis, this study was the first one to use large datasets of VLBW infants from well-established national neonatal databases from Japan and Brazil to compare the neonatal outcomes between both countries. One problem associated with the use of such neonatal databases is the number and definition of variables available in each database to make comparison between countries. However, we were able to overcome these difficulties by adjusting individual patient level data, and by incorporating perinatal and neonatal factors in the analyses.

Notwithstanding, there are some limitations to this study that merit consideration. Firstly, the selected analysis period was more than 5 years ago, therefore this could have led to under- or overestimated data analysis compared to the present day. On the other hand, as the article shows robust data from many hospitals, it leads us to infer that the results found would not differ from the current scenario. Secondly, this was a retrospective observational study. As the definition of the variables may vary between countries, we had to reach a consensus on the criteria used by the two networks to adjust the definitions of the outcome. However, both networks had used a standardized form developed by investigators similar to those of the international neonatal database of VLBW infants from Vermont Oxford Network.^11^ Consequently, we only observed minor differences in some definitions. Thirdly, this study made a comparison between a developed and a developing country, therefore there could be other culture- and context-specific variables, such as socioeconomic status, that may influence the outcomes. As we had no access to information about these variables, it was impossible to make such adjustments in the final model. Fourthly, this was a secondary data analysis based on the availability of retrospectively collected data, therefore the severity of the morbidities could not be accounted for in our comparison.

Despite the limitations of the study, our analyses identified factors that increase the odds of in-hospital neonatal death in Brazil, most of which were related to LOS. Our findings highlighted the importance of clinical practices related to the management of care for VPT and VLBW infants. Efforts are needed to explore the factors associated with LOS and design interventions to reduce the incidence and deaths associated with LOS in Brazil. Further studies are required to provide reliable data and scientific evidence to support guideline development for neonatal health management, especially in developing countries, to guide not only health care professionals but policymakers in areas where further improvement is needed to reduce the disparities in neonatal outcomes among countries.
